# An Overview of the Use of Precision Population Medicine in Cancer Care: First of a Series

**DOI:** 10.7759/cureus.37889

**Published:** 2023-04-20

**Authors:** Johnny Yang, Mary R Nittala, Alexander E Velazquez, Vedanth Buddala, Srinivasan Vijayakumar

**Affiliations:** 1 Radiation Oncology, University of Mississippi Medical Center, Jackson, USA; 2 Medicine, University of Mississippi Medical Center, Jackson, USA

**Keywords:** multi-cancer early detection, artificial intelligence, big data, genomic medicine, precision medicine, precision population medicine

## Abstract

Advances in science and technology in the past century and a half have helped improve disease management, prevention, and early diagnosis and better health maintenance. These have led to a longer life expectancy in most developed and middle-income countries. However, resource- and infrastructure-scarce countries and populations have not enjoyed these benefits. Furthermore, in every society, including in developed nations, the lag time from new advances, either in the laboratory or from clinical trials, to using those findings in day-to-day medical practice often takes many years and sometimes close to or longer than a decade. A similar trend is seen in the application of “precision medicine” (PM) in terms of improving population health (PH). One of the reasons for such lack of application of precision medicine in population health is the misunderstanding of equating precision medicine with genomic medicine (GM) as if they are the same. Precision medicine needs to be recognized as encompassing genomic medicine in addition to other new developments such as big data analytics, electronic health records (EHR), telemedicine, and information communication technology. By leveraging these new developments together and applying well-tested epidemiological concepts, it can be posited that population/public health can be improved. In this paper, we take cancer as an example of the benefits of recognizing the potential of precision medicine in applying it to population/public health. Breast cancer and cervical cancer are taken as examples to demonstrate these hypotheses. There exists significant evidence already to show the importance of recognizing “precision population medicine” (PPM) in improving cancer outcomes not only in individual patients but also for its applications in early detection and cancer screening (especially in high-risk populations) and achieving those goals in a more cost-efficient manner that can reach resource- and infrastructure-scarce societies and populations. This is the first report of a series that will focus on individual cancer sites in the future.

## Introduction and background

The precision medicine (PM) initiative describes precision medicine (PM) as an “emerging approach for disease treatment and prevention that considers individual variability in genes, environment, and lifestyle for each person” [1]. This approach allows doctors and researchers to create optimal treatments and prevention strategies for diseases specific to groups of people. This contrasts with a one-size-fits-all approach and allows for individualized prevention, disease screening, and treatment plans.

Given that PM is becoming vital to quality patient care, this paper aims to define the current literature on PM and how it relates to overall patient care. Serving as the introduction to a series of about 10 cancer disease site-oriented reviews, this report of the current understandings of general PM will provide a natural segue for a thorough discussion of PM as it relates to a variety of cancers (i.e., breast, cervical, lung, brain, head/neck, pediatric, prostate, renal, lymphoma/leukemia, liver, stomach, colorectal, and pancreatic), legislation, and radiomics/artificial intelligence (AI). More importantly, this series of reviews could elucidate the methods of achieving optimal patient care and disease screening and prevention.

**Figure 1:**
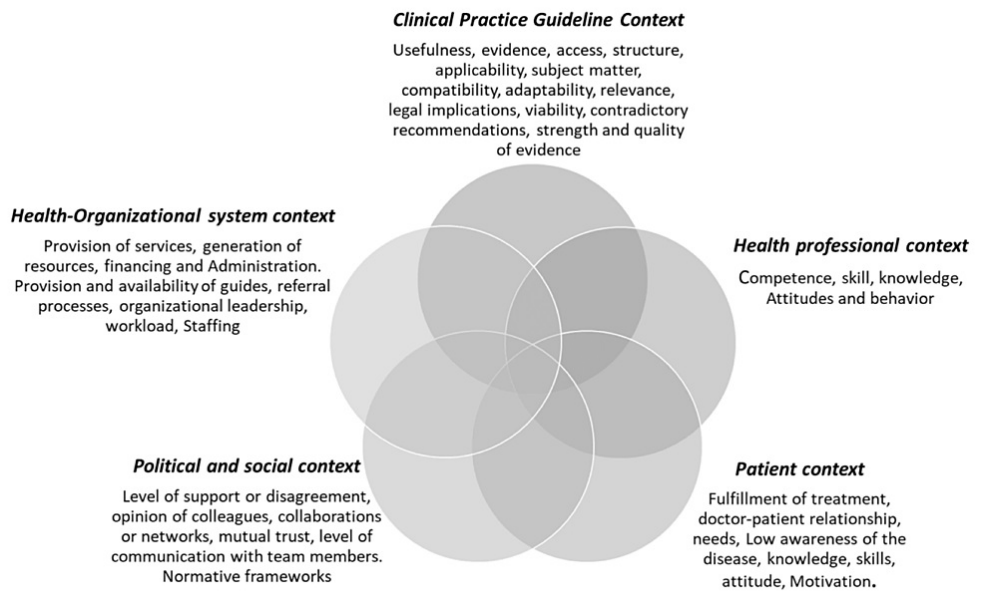
Contexts to explore barriers and facilitators for clinical practice guidelines. This image is reproduced from Correa et al. [2] and is available via Creative Commons Attribution 4.0 International License.

There is a significant gap in applying research findings to actual clinical and health practices. Multiple domains lead to these barriers (see Figure 1) [2].

The details of how to overcome these barriers in each domain are beyond this paper’s scope. However, in each one of these domains, a constant factor that emerges is the knowledge about the details of the subject involved. Defining the issue and synthesizing the knowledge from an interdisciplinary point of view can help overcome these barriers. The paper aims to synthesize the knowledge from an interdisciplinary point of view to show the need for redefining the “breadth” of PM from its narrow genomic medicine (GM) = PM perspective to a much broader vision of its benefits in the public/population health (PH) dimension.

## Review

Search databases and search strategy

This is a narrative review where databases such as Medical Literature Analysis and Retrieval System Online (MEDLINE)/PubMed and Google Scholar were used for the literature search between 1997 and 2022. The databases were screened with the individual medical subject heading keywords and key term combinations, including “precision medicine,” “genomic medicine,” “big data,” “public health,” “electronic health records,” “artificial intelligence,” and “cancer detection.”

Inclusion/exclusion criteria

Studies only in English from 2010 to 2022 were included, with no other specific filters being used. Commentaries, letters to the editor, and unpublished reports were excluded.

What is the “right definition” of precision medicine?

PM’s inception and clinical applications significantly precede the definition of PM itself. Though healthcare has employed PM for years and while the concept is not new, elucidating its purpose is novel and will be thoroughly discussed in this paper. Before its definition, PM was employed by healthcare providers, researchers, and politicians to improve the accuracy of predicting disease treatments, management, and measures depending on the differences between individuals, such as those between various ethnicities [3]. Providing individual-based care, such as matching ABO blood transfusions and lens customizations, reflects PM’s applications precisely [4]. Our current understanding of the PM concept and its associated clinical value has provided many opportunities to apply it to many challenges in healthcare [5].

President Obama’s “precision medicine initiative” was an invaluable drive to increase PM’s momentum [6]. PM is a relatively new disease prevention and therapy method with a valuable ability to consider and integrate varying genetic, environmental, and social factors [7]. Owing to that, a significant fraction of public health funding has been devoted to the current advancements in PM research, such as genetic testing, screening, and counseling. As a result, PM is constantly questioned and challenged to determine its utility. Critics’ primary concern is whether PM yields health improvements for the general population or can the funding and efforts be better allocated toward conventional public health measures [8]. To understand PM, defining it will aid in clarifying these conflicting perspectives and provide a balance between PM and public health/population health (PH).

In determining the appropriate definition for PM, it is essential to consider biological and social factors, such as socioeconomic status, the level of education, the cultural outlook of medicine, environmental aspects, and lifestyle habits. Participation from diverse backgrounds is critical to generate the most benefit when employing PM. In addition, providing the proper healthcare at the appropriate time for individuals and populations requires some considerations. However, it is not limited to economic capabilities, genetic predispositions based on different ethnicities, religious beliefs, and behavioral tendencies based on location and age [9]. Understanding and applying social factors, combined with genomic advancements, may reveal the potential role of PM in reducing current healthcare disparities that burden underrepresented communities.

**Figure 2:**
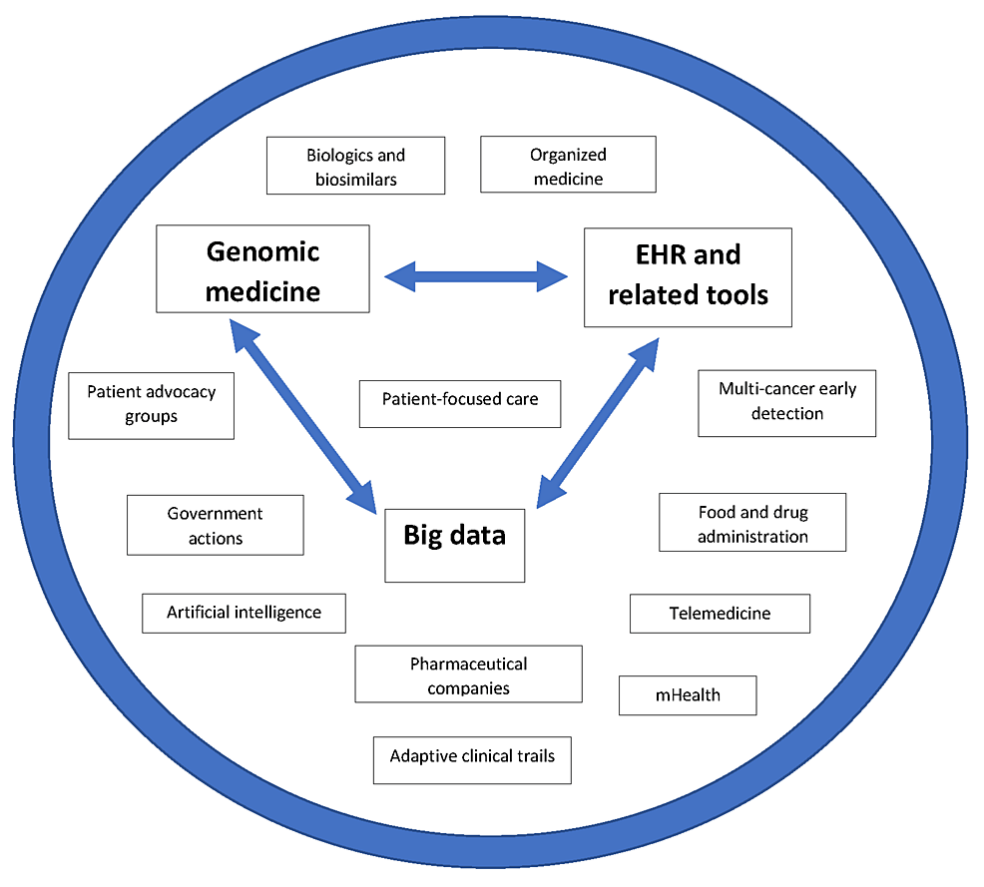
The components of precision medicine. This image is a simplified depiction from the authors of this paper. EHR, electronic health records; mHealth, mobile health

As a few examples of the current PM methods implemented focusing on PH, the Human Genome Project (HGP), big data initiative, and electronic health records (EHR) consistently evolve and alter therapeutic approaches [5,10-15]. When these methods are combined with GM, clinicians can begin to overcome the challenges of providing the appropriate therapy or preventative measure for a particular case for a variety of pathologies such as chronic diseases and cancers [5,16,17]. For instance, by employing GM in patients diagnosed with cervical cancer, Pinheiro et al. associated HPV35 A2 sub-lineage with cancer in African American (AA) individuals. This association supports the disproportionate prevalence of all-invasive cervical cancer [16]. In addition to GM’s role in highlighting a genetic influence, PM methods allow researchers and clinicians to optimize care for high-risk groups further. They, therefore, assess outcomes more accurately and individually, such as when employing big data initiatives [5,17]. Figure 2 is the schematic representation of the PM components, the elements that will shape disease care and prevention in the future.

**Figure 3:**
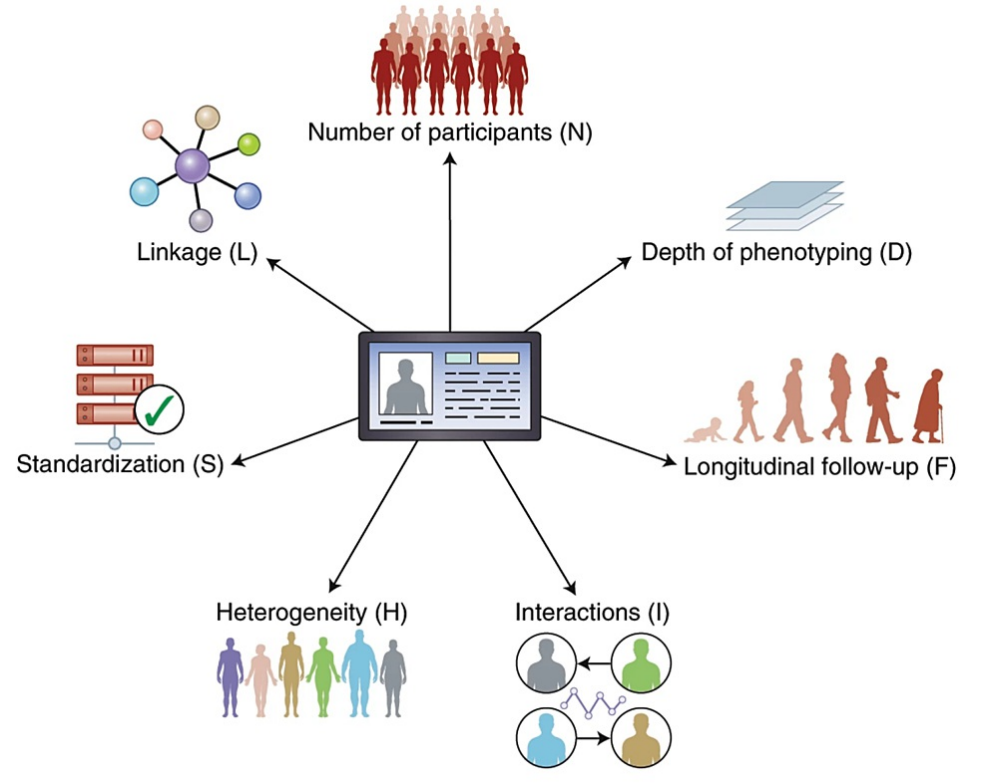
The different axes of health data. The complexity of large health datasets can be represented by distinct axes, each encompassing a quantifiable property of the data. This image is reproduced from Shilo et al. [12], and permission was obtained from the licensed content publisher Springer Nature.

By utilizing a broader classification for PM and expanding to include more methods and factors incorporated into PM, more accurate data strategies can be developed to treat patients more efficiently, prevent poor health conditions, and alleviate healthcare disparities. There is value in expanding coverage for prevention, early detection, and advancement; PM’s diagnostic tools and technologies, such as smartphones and smartwatches, would aid the synchronization of PH goals. Figure 3 depicts the quantitative properties of the big data initiative [12].

Is precision medicine the same as genomic medicine?

We posit that PM includes GM, but PM is not the same as GM. PM is significantly more encompassing than GM. Clarifying this definition is essential to take advantage of many new PH developments and advances in the past decade with the ultimate goal of improving patient care in a holistic, cost-effective, and time-efficient manner; concurrently increasing access beyond populations of higher socioeconomic status will better serve countries and those that are limited by resources.

**Figure 4:**
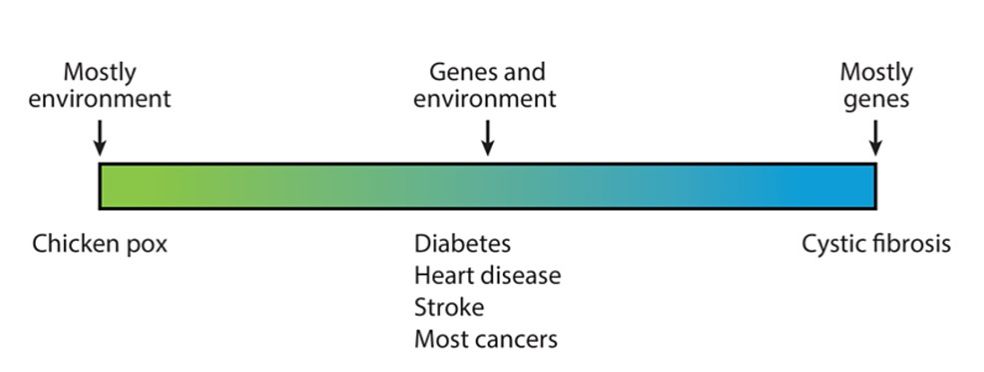
The contributions of genes and social environment to health outcomes. Genetic contributions to health occur along a continuum. The outcome in rare genetic diseases is determined primarily by genes; the outcome in other diseases, such as chicken pox, is determined primarily by the social environment. In the middle are diseases with a varying mix of genetic and environmental contributors, such as diabetes, heart disease, stroke, and most cancers; these conditions represent the major disease burdens in the United States. This image is reproduced from Burke [19] and is available via Creative Commons Attribution 4.0 International License.

The definitions of PM and GM are essential when discussing the differences. As a diagnostic and therapeutic tool for patient care, GM is a medical discipline incorporating an individual’s genomic information into treatment plans [18]. Figure 4 exhibits a continuum for the role of environmental and genetic contributors in health outcomes [19].

Assessing specific genes and their interactions can dramatically increase the effectiveness of predicting an individual’s disease course and developing personalized treatment plans. For instance, in testing for the compatibility between an individual and a medication, varying genetic mutations can be compared with known adverse drug reactions and positive patient outcomes [18,20].

As an example of GM, thiazide diuretics serve as a first-line medication for AAs with hypertension [20,21]. In a study by Armstrong et al. [20] that analyzed the genetic contributors to the adverse metabolic effects of a thiazide diuretic, chlorthalidone, in AAs, eight and nine variants were associated with blood pressure and fasting glucose response, respectively. Despite thiazide’s beneficial blood pressure response, the detrimental glucose response yields clinical importance. The authors suggested that employing this genomic knowledge in patient care would significantly reduce and/or prevent adverse cardiovascular events with a caveat to consider adverse glucose effects. Armstrong’s work highlights an essential perspective of GM: GM alone is a field of clinical importance and research that can grow. Despite thiazide therapy demonstrating the value of GM for improving AA patient outcomes, these studies also illustrate a critique of a “one-size-fits-all” treatment approach for a single race; more subtle differences can exist between individuals of the same race and are worth further stratifying, which would require additional research and investment [18]. Despite the complexity of GM approaches, their ability to improve patient care warrants future investments.

Regarding PM’s broader field, which includes the subset of GM, PM can be more impactful by considering the previously mentioned social factors. Consequently, researchers and clinicians are provided with an increased population testing size and an opportunity to improve patient care by employing big data initiatives and epidemiological interventions [22-24]. Therefore, we suggest that while GM can enhance a small fraction of the general population, PM’s broader scope focusing on PH goals can impact a higher proportion of the general population, thereby elevating prognostic, diagnostic, and therapeutic approaches to the next level [25]. However, as an important caveat, the applications of PM components, including GM and PH goals, require a concerted and synergistic approach to yield success.

In the scope of PM, improvements in diagnostic methods include increased practical applications of biomarkers, molecular testing, and genetic sequencing to identify high-risk individuals. While biomarkers have previously been employed for identifying pathologies (heart disease and specific types of cancers) with genetic associations, improved applications for the utilization of PM methods, such as big data initiatives, offer the opportunity to identify correlations between specific social/environmental variables with early identification of high-risk individuals [8]. For example, the genetic sequencing of patients at high risk for breast cancer in the United States has identified BRCA1 and BRCA2 as strong genetic markers. Approximately 55%-65% of individuals with BRCA1 and about 45% of females with BRCA2 will develop breast cancer by the age of 70 [8,26,27]. Given that these specific mutations additionally increase the likelihood of ovarian cancer, molecular testing is invaluable in reducing the burden on these patients. However, cancer reduction methods for GM are limited because genetic sequencing and identification constitute approximately 5%-10% of breast cancer cases [25]. This limitation encourages the inclusion of PH-focused PM principles and strategies such as telemedicine, health policy, and data dissemination, all for representing a larger population and subsequently improving treatment strategies [28-30].

Why is the current definition of precision medicine too narrow?

Despite the apparent differences between PM and GM, the two are often grouped as the same in practice. For example, the American Cancer Society suggested that PM is how clinicians can provide patient care based on a patient’s genetic background [31]. Regarding the emerging PM field, the heavy emphasis on GM strategies creates logistical problems by potentially mislabeling PM and prevents the development of PM research, preventative tools, and therapeutics.

High ceiling for improvements in genomic medicine

**Figure 5:**
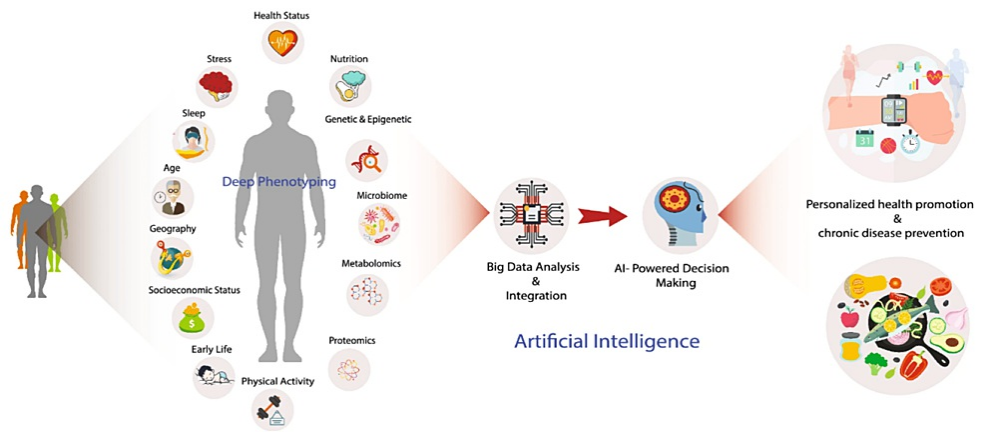
Precision medicine in the era of artificial intelligence (AI): implications in chronic disease management. Deep phenotyping and artificial intelligence for health promotion and chronic disease prevention. Deep phenotyping provides an entire molecular profile of an individual’s physiological status. When longitudinally tested, the pathways can be tracked to identify the transformation from a health to a disease. Various omics technologies along with other physiological measurements will be used to molecularly characterize an individual’s risk for disease. The further implementation of a systems approach to big data analysis and integration will provide a platform for machine learning and artificial intelligence in clinical decision-making for early disease risk identification and prevention. This image is reproduced from Subramanian et al. [33] and is available via Creative Commons Attribution 4.0 International License.

**Figure 6:**
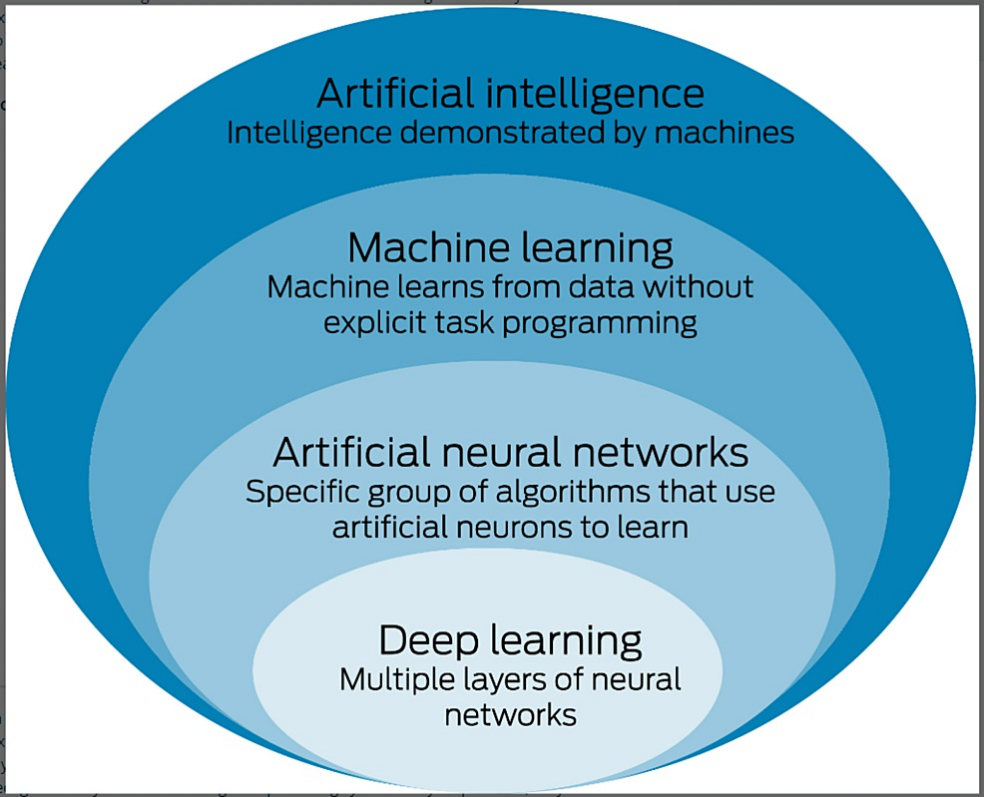
Relationship between artificial intelligence and its subtypes. This image is reproduced from Scheetz et al. [34], and permission was obtained from licensed content publisher AMPCo Pty Ltd.

GM’s high costs and limited data currently serve as the primary challenges preventing maximal PM development. Works by Walsh et al. suggested that GM costs can be a low-value investment for diseases with low penetrance genes (i.e., rare diseases or conditions) [32]. With rarer diseases and conditions, new GM research and technology may not be worth the cost, bringing into question if there are less expensive alternatives that can provide similar outcomes compared to GM. Additionally, improvements in GM possess the potential to improve disease treatment and personalized care drastically. Still, they are not always viable due to the inherent limitations of diagnostic technology and data, such as artificial intelligence and data protection/privacy. Therefore, before investing in GM, the benefits must be critically considered with the high ceiling of high cost, artificial intelligence, and data protection/privacy. Figure 5 depicts the application of artificial intelligence to improve health outcomes and disease prevention, and its subsets are depicted in Figure 6 [33,34].

Unequal access to genomic medicine

Funding GM research also serves as an obstacle for developing and resource-limited countries. Since most governments cannot fund the ideal full-scale investments from inception to translationally incorporating genomic tools, the developments of GM are disproportionately spread among countries [35]. Furthermore, the high costs of GM research prevent underdeveloped countries from implementing effective PM strategies in clinical use, further exacerbating healthcare disparities. Therefore, to promote the maximum benefit for the general population, varying policies should be implemented with a more in-depth consideration of each country’s ability to invest in GM development.

Limited diversity in genomic data

Most genomic data sources lack diversity since they are derived from a limited database of predominantly European populations, further exacerbating disparities between countries employing PM [19]. Despite some advances in promoting diversity in GM, additional research and genetic data from different populations and ethnicities are required before GM can benefit as a beneficial tool for all populations [36,37].

Although big data initiatives are undervalued clinically due to an unrealized potential by the healthcare industry, they can be employed with proper research and implementation to formulate more accurate data-driven treatments and strategies [38]. According to a systematic review by Battineni et al., telemedicine has improved cardiovascular disease care by impacting the monitoring by virtual meetings, mediating emergency room visits, and ultimately lowering the cost of care, particularly for developing countries [39]. For PM applications in a clinical setting, the significant emphasis on GM research inadvertently fosters cost and diversity issues. With the inclusion of different methods beyond GM, PM has the potential to provide personalized care to a more generalized population. Thus, the definition of PM is too narrow and must include these varying strategies.

What should be the real definition of precision medicine?

Considering our previous discussion on the limitations of PM’s narrow definition, PM’s definition should be expanded to include a more realistic and impactful description, one that regards the population level and the population’s individuals. Through GM-focused strategies such as improving diagnostic methods, PM preventatively cares for high-risk populations by detecting early risk onsets such as cervical, lung, and prostate cancers [40-42]. However, PM should be starkly differentiated from GM to investigate, access, and implement the benefits of PM beyond that of GM toward the greater goal of serving more populations. To promote PH as a priority in PM discussions, a natural first step would be encouraging an intentional change in diction used in conversations and by clinicians, researchers, and educators; however, a larger-scaled impact can be observed by also encouraging policymakers to adopt a similar change, which may be more likely to occur with the help of conversations brought forth by clinicians, researchers, and educators in a concerted effort [25]. Positing that the true PM definition should combine the advances of GM (i.e., improved diagnostic tools) and big data initiatives, telemedicine, and PH goals (i.e., big data screening), we offer and coin a novel term “precision population medicine” (PPM). Through PPM, we hope that PPM’s considerations for biological markers and environmental/social factors within a population will better provide care on an individual and population basis.

What will be the benefits of an expanded definition?

Refining PPM to differentiate GM and PH goals can profoundly impact healthcare by allowing future developments and conversations to extend into both areas of GM and PH. Consequently, both can be maximally investigated and clinically employed to provide more personalized preventative strategies and therapy for individuals and populations [25]. Recent works discussing liquid biopsies with breast cancer, genomic sequencing with cervical cancer, and multi-cancer early detection (MCED) demonstrate PPM’s potential [8,16,42-50].

Combining (diagnostic) imaging, artificial intelligence, genomic medicine, big data, and other epidemiological tools in precision population medicine: The emergence of radiogenomics (RG)

Radiogenomics (RG) [51] is defined with some variations (Table 1) [52-56]. Suffice it to say that RG will play an important role in PPM.

**Table 1:** Defining radiogenomics.

Item number	Definition	Source	Comment
1	Radiogenomics (RG) is a novel research field focusing on establishing associations between radiological features and genomic or molecular expression in order to shed light on the underlying disease mechanisms and enhance diagnostic procedures toward personalized medicine.	Trivizakis et al. [52]	We agree with this wholeheartedly; the application of these concepts will lead to a “broadening” of RG’s applications (see next comment below).
2	Radiogenomics designates a scientific field that addresses possible associations between genetic germline alterations and normal tissue toxicity after radiotherapy. The ultimate aim of this research is to establish a gene-based predictive test for normal tissue radiosensitivity.	Andreassen et al. [53]	This may be a too narrow a definition; RG should be able to play a role in all domains of cancer care: prevention, screening early detection, diagnosis, prognostication, treatment choices, dose escalation/de-escalation, prediction of response, early detection of recurrence, etc.
3	Radiogenomics has emerged as a state-of-the-art science in the field of individualized medicine. RG combines a large volume of quantitative data extracted from medical images with individual genomic phenotypes and constructs a prediction model through deep learning to stratify patients, guide therapeutic strategies, and evaluate clinical outcomes.	Shui et al. [54]	We agree. The only addition we would like to add is to expand RG’s applications beyond cancer treatment and management to other domains as well, as we outlined in our previous comment under number 2.
4	Radiomics, which extracts large volumes of quantitative data from digital images and amalgamates these together with clinical and patient data into searchable shared databases, potentiates radiogenomics, which is the combination of genetic and radiomic data. Radiogenomics may provide voxel-by-voxel genetic information for a complete, heterogeneous tumor or, in the setting of metastatic disease, a set of tumors and thereby guide tailored therapy.	Pinker et al. [55]	We agree. Again, we would like the definition to be broadened further (see above).
5	Radiogenomics is the extension of radiomics through the combination of genetic and radiomic data. Because genetic testing remains expensive, invasive, and time-consuming and thus unavailable for all patients, RG may play an important role in providing accurate imaging surrogates, which are correlated with genetic expression, thereby serving as a substitute for genetic testing.	Lo Gullo et al. [56]	We agree. However, with the emergence of liquid biopsies, the cost of and duration to obtain genetic testing is/will be decreasing. So, the usefulness of RG can be optimized by applying it to higher-risk populations/in more selected circumstances, in other words using it in a more “nuanced” way.

RG is predicted to get into daily cancer care vocabulary [53,57] sooner than later. Andreassen et al., in their recent comprehensive review of the role of RG in cancer care, conclude that RG will influence every facet of cancer care, from early detection, diagnosis, clinical management, and surveillance (see Table 1). They also point out many advantages of RG in cancer care: (a) RG makes the global tumor information available compared to infinitesimal data from a biopsy specimen. (b) This holistic tumor information is further enhanced by unrevealed spatial (potential biological) heterogeneity variation details. This can help predict prognosis and the ability to titrate the treatment (or, from this paper’s point of view, “titrate” cancer risk predictability, e.g., in a precancer lesion) based on an individual’s biology. (c) RG will be lower in cost than conventional genome sequencing [53].

**Figure 7:**
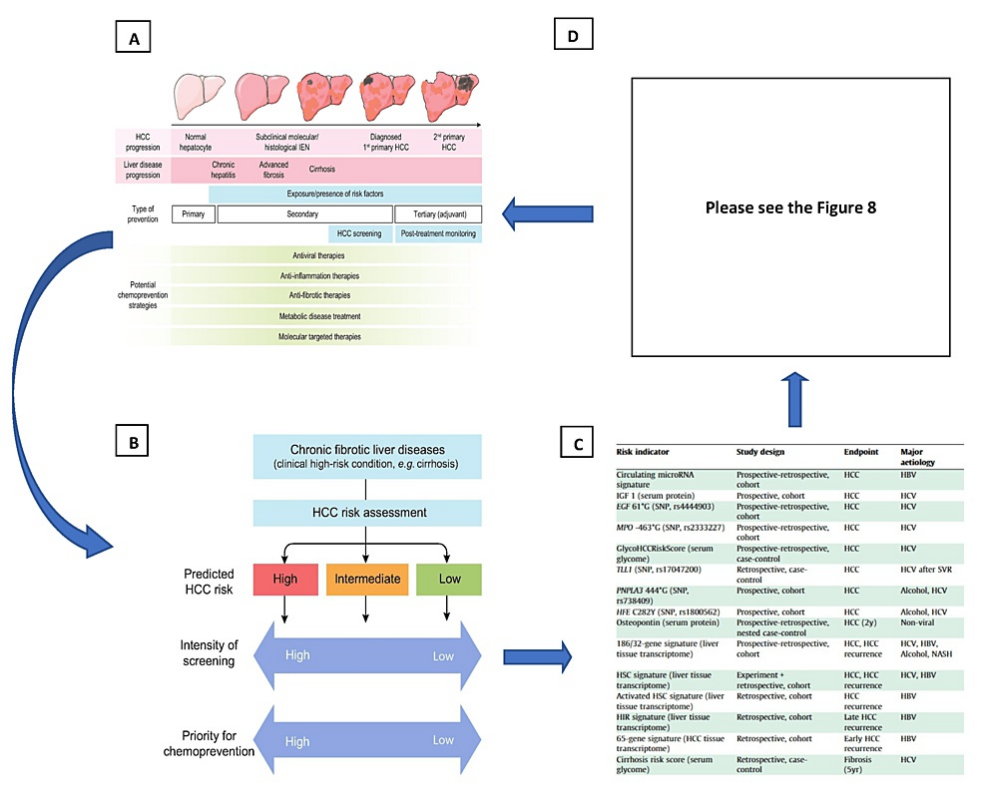
Radiogenomics in precision population health: the future envisioned. (A) Hepatocellular carcinoma, preventive interventions in the natural history of HCC development in progressive fibrotic liver diseases. (B) Individual risk-based tailored hepatocellular carcinoma screening and chemoprevention. (C) Clinical hepatocellular carcinoma/fibrosis risk indicators. (D) Molecular targets of potential hepatocellular carcinoma chemoprevention therapies (the illustration in panel D is unclear, so we added a separate figure: Figure 8). This image is reproduced from Fujiwara et al. [58], and permission was obtained from the licensed content publisher Elsevier. HCC, hepatocellular carcinoma, IEN, intraepithelial neoplasia; HBV, hepatitis B virus; HCV, hepatitis C virus; IGF 1, insulin-like growth factor 1; SVR, sustained virologic response; NASH, non-alcoholic steatohepatitis; EGF, epidermal growth factor; MPO, myeloperoxidase; SNP, single nucleotide polymorphism; TLL1, tolloid-like protein 1; HSC, hepatic stellate cell; HIR, hepatic injury and regeneration

This topic’s potential is so vast that only one disease site will be addressed here as an illustration: hepatocellular carcinoma (HCC). Viral or metabolic etiologies lead to fibrotic changes in the liver parenchyma, increasing the risk of developing HCC [58]. Fujiwara et al. point out that current “one-size-fits-all” practice guidelines must be revised and cost-efficient to significantly impact on early diagnosis, chemopreventive success, or outcome improvement [58]. Fortunately, many new GM-based biomarkers are emerging in HCC [59]. Johnson et al. point out that cell-free (cf) DNA, cfRNA, cf mitochondrial DNA, cf viral DNA, and extracellular vesicles from liquid biopsies can be useful potential biomarkers in HCC. by using these new GM-based biomarkers, high-risk populations can be more easily identified than alpha-fetoproteins and ultrasound-based screening, including among resource-scarce populations [58,60]. Furthermore, these liquid biopsy-based biomarkers can be combined with new AI-driven imaging models to identify high-risk populations and use chemoprevention applications to diagnose/prevent/improve outcomes in HCC [61]. These concepts are further expanded in Figure 7 [58].

**Figure 8:**
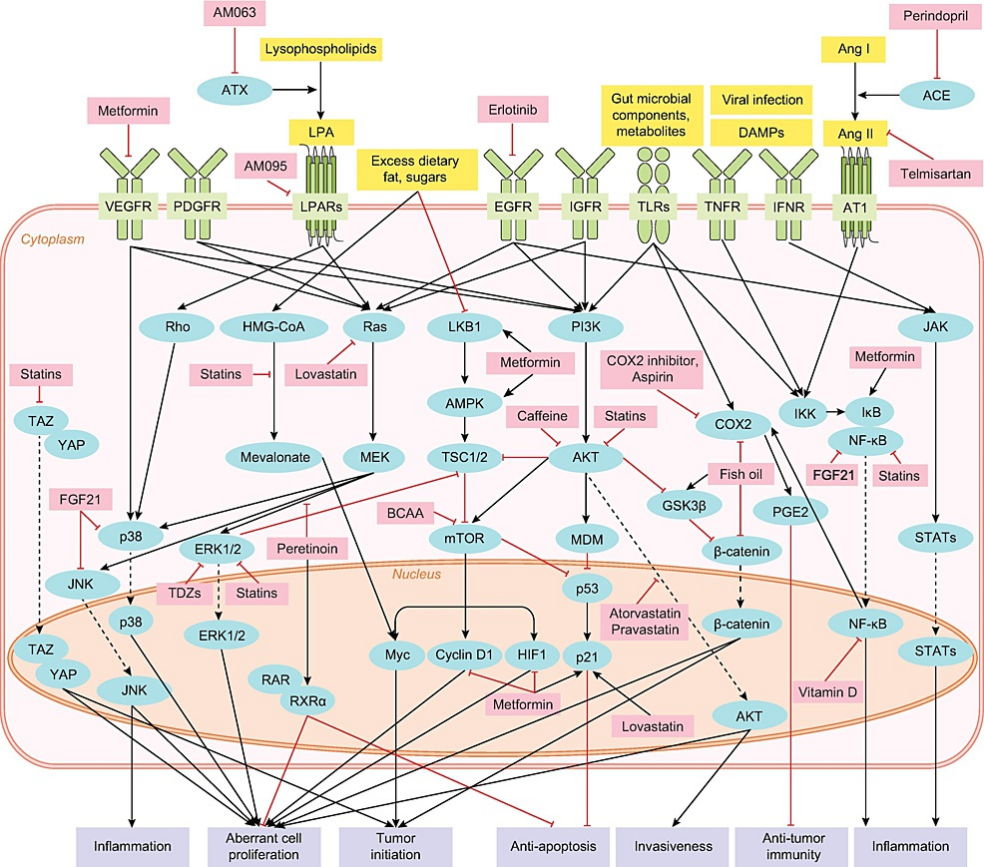
Molecular targets of potential hepatocellular carcinoma (HCC) chemoprevention therapies. Intra- and extracellular targets of potential HCC chemopreventive therapies are summarized. Solid line with arrowhead or bar: activation or inhibition; dotted line with arrowhead: translocation between intracellular compartments. This image is reproduced from Fujiwara et al. [58], and permission was obtained from the licensed content publisher Elsevier. ACE, angiotensin-converting enzyme; AMPK, adenosine monophosphate-activated protein kinase; Ang, angiotensin; ATX, autotaxin; AT1, angiotensin type 1 receptor; BCAA, branched chain amino acid; COX2, cyclooxygenase 2; DAMPs, damage-associated molecular patterns; EGFR, epidermal growth factor receptor; ER, endoplasmic reticulum; ERK, extracellular signal-regulated kinase; FGF21, fibroblast growth factor 21; GSK3, glycogen synthase kinase 3; HIF, hypoxia inducible factor; HMG-CoA, 3-hydroxy-3-methyl-glutaryl-coenzyme A; IFNR, interferon receptor; IGFR, insulin-like growth factor 1 receptor; JAK, Janus kinase; JNK, c-Jun N-terminal kinase; LKB1, liver kinase B1; LPA, lysophosphatidic acid; LPAR, lysophosphatidic acid receptor; MDM, mouse double minute; mTOR, mammalian target of rapamycin; NF-κB, nuclear factor-kappa B; PDGFR, platelet-derived growth factor receptor; PGE2, prostaglandin E2; PI3K, phosphoinositide 3-kinase; RAR, retinoic acid receptor; ROS, reactive oxygen species; RXR, retinoid X receptor; STAT, signal transducers and activator of transcription; TDZ, thidiazuron; TLRs, Toll-like receptors; TNFR, tumor necrosis factor receptor; TSC, tuberous sclerosis complex; VEGFR, vascular endothelial growth factor receptor; YAP, Yes-associated protein; TAZ, transcriptional coactivator with PDZ-binding motif; MEK, mitogen-activated protein kinase enzyme; AKT, serine/threonine kinase; IKK, IkB kinase complex; IκB, inhibitor of nuclear factor-kappa B

Figure 7 is a “collage” of selected tables and figures from Fujiwara et al. [58] that synthesizes the concepts outlined by them, as well as from others [59,61-63]. Panel D from Figure 7 is further clearly represented in Figure 8.

PPM can decrease disease prevalence and increase disease prevention by assessing high-risk individuals. Considering a previous discussion of a breast cancer study by Ramaswami et al., advancements in screening for biomarkers (i.e., BRCA1/2) can be groundbreaking [8]. Still, the major limitation (accounting for only 10% of cases) suggests that investments in areas beyond GM are necessary to combat leading cancer incidence in females. Although mammography screening is the most common diagnostic tool for breast cancer, it is not widely accessible in rural areas due to limitations such as trained physicians. Therefore, mammography poorly reflects PPM ideas. Additionally, approximately 30% of breast cancer patients in sub-Saharan Africa present after the conventional age of initial screening (50 years), suggesting the minimal screening utility in developed countries [8,43]. Other issues include the high rate of false positives, patient distress, and decreased quality of life. While these points collectively suggest that screening is a poor tool for various populations, they also provide motivation and potential for pursuing a more-inclusive PPM approach.

**Figure 9:**
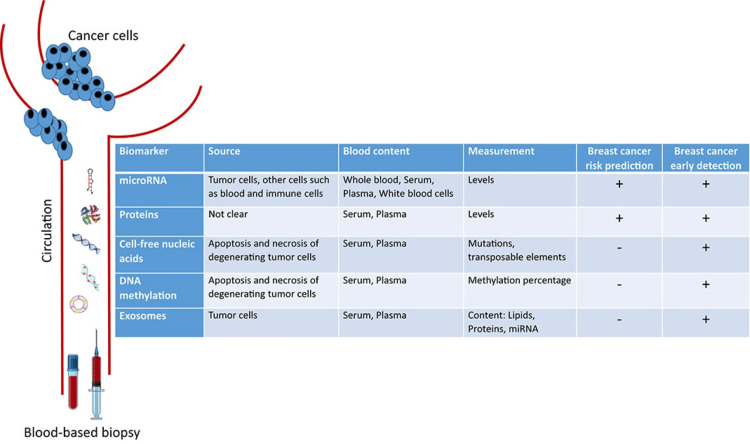
Peripheral blood-based biopsy for breast cancer risk prediction and early detection. This image is reproduced from Nassar et al. [43] and is available via Creative Commons Attribution 4.0 International License.

As depicted in Figure 9, clinicians can alternatively employ liquid biopsies to examine biomarkers in high-risk healthy individuals and large and ethnically diverse populations [43]. Compared to mammography, this more meaningful intervention can alter the landscape for diagnosing breast cancer with early detection and accurate risk prediction; addressing its major limitation of lacking popularity requires the support of policymakers to provide more funding and resources for research and development.

**Figure 10:**
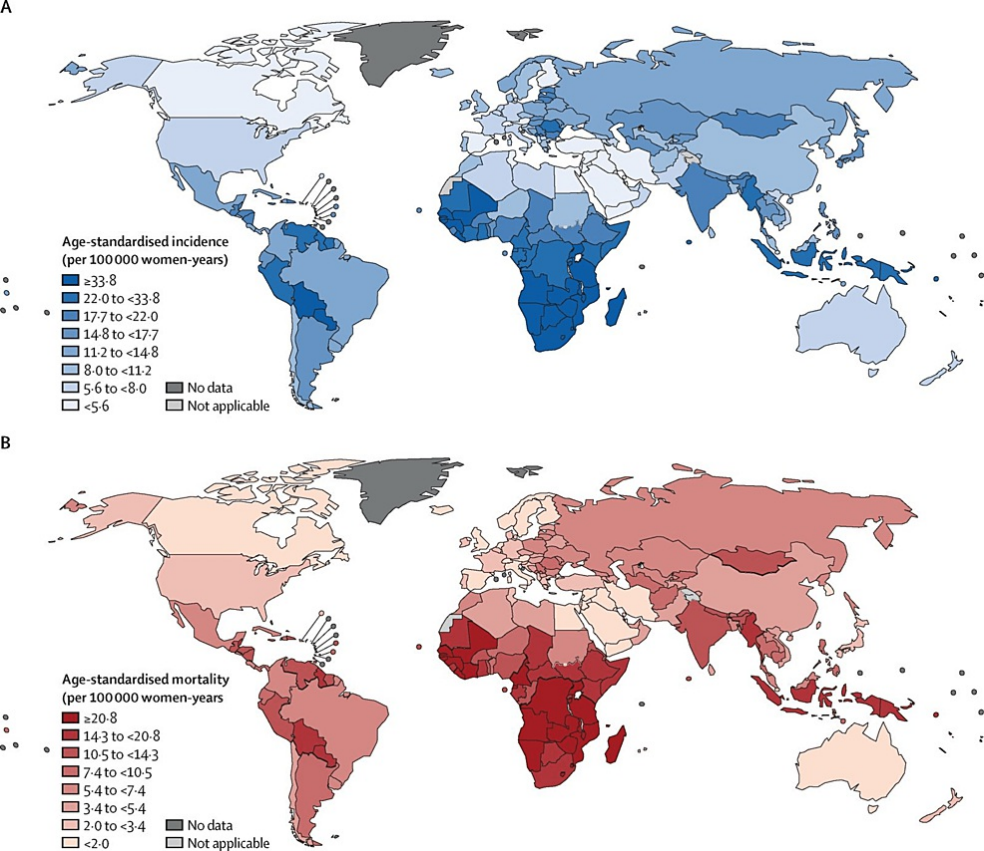
Age-standardized incidence (A) and mortality rates (B) of cervical cancer by country in 2020. Data are from the GLOBOCAN database, collated by the International Agency for Research on Cancer and hosted by the Global Cancer Observatory. This image is reproduced from Singh et al. [45], and permission was obtained from the licensed content publisher Elsevier.

With an estimated regression rate of 60%, cervical cancers are associated with human papillomavirus (HPV) infections; therefore, many preventive medicine efforts have been dedicated to HPV [42,44]. Figure 10 depicts age-adjusted incidence and mortality rates of cervical cancer worldwide in 2020 [45].

In a study by Pinheiro et al. including sub-Saharan AA females, exposure to the A2 sub-lineage of HPV35 was associated with higher rates of invasive cervical cancer (ICC), where HPV35 is 2% and 10% of ICC in the world and only sub-Saharan Africa, respectively. Using the National Cancer Institute data, the authors successfully differentiated benign from high-grade infections in African and non-African regions. More importantly, the study highlighted that PPM provided the opportunity to develop more targeted approaches for populations that can regard people with a similar race or region [16].

**Figure 11:**
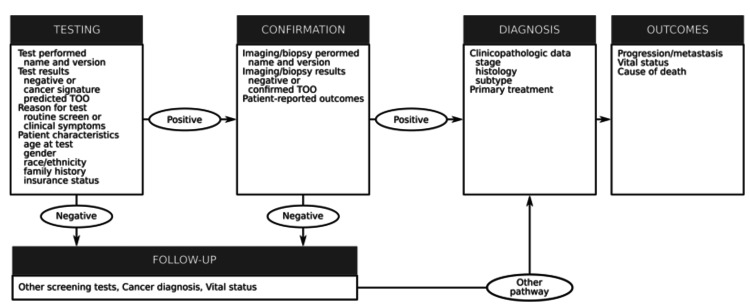
Schematic of the components of a data registry to track the utilization and outcomes of patients receiving MCED tests. This image is reproduced from Etzioni et al. [48], and permission was obtained from licensed content publisher Oxford University Press. TOO, tissue of origin; MCED: multi-cancer early detection

Representing another application of PPM, MCED employs free circulating DNA to detect cancers, especially those that lack guideline screening [46,48-50]. This tool considers the frequency of cancer types, stage/grade, and symptomatic versus asymptomatic factors toward developing trends. As a new modality to care for populations using distinct components in blood samples, MCED allows populations such as rural ones to overcome limited access to healthcare. Figure 11 depicts a general schematic for MCED utilization and could aid new registries [48].

Furthermore, increased emphasis on precision medicine and advocacy by policymakers are required to employ new emerging technologies such as Galleri and CancerSEEK to detect multiple cancers with one blood sample with an impressively low 5%-10% false-positive rate [46]. A similar benefit can be observed with income considerations when examining whether MCED is employed in developing countries [50].

Additionally, the massive improvements in biological data acquisition have allowed the analysis of population and individual samples. Environmental and biological data, on multiple scales, can elucidate invaluable trends enabling earlier cancer detection and improving quality of life. This pairing would ideally foster a more lasting improvement in healthcare [25,30]. PM and GM share a symbiotic relationship where GM is essential to creating more personalized therapeutic strategies. PM furthers this ability with the addition of many other tools to create more representative strategies for populations.

**Figure 12:**
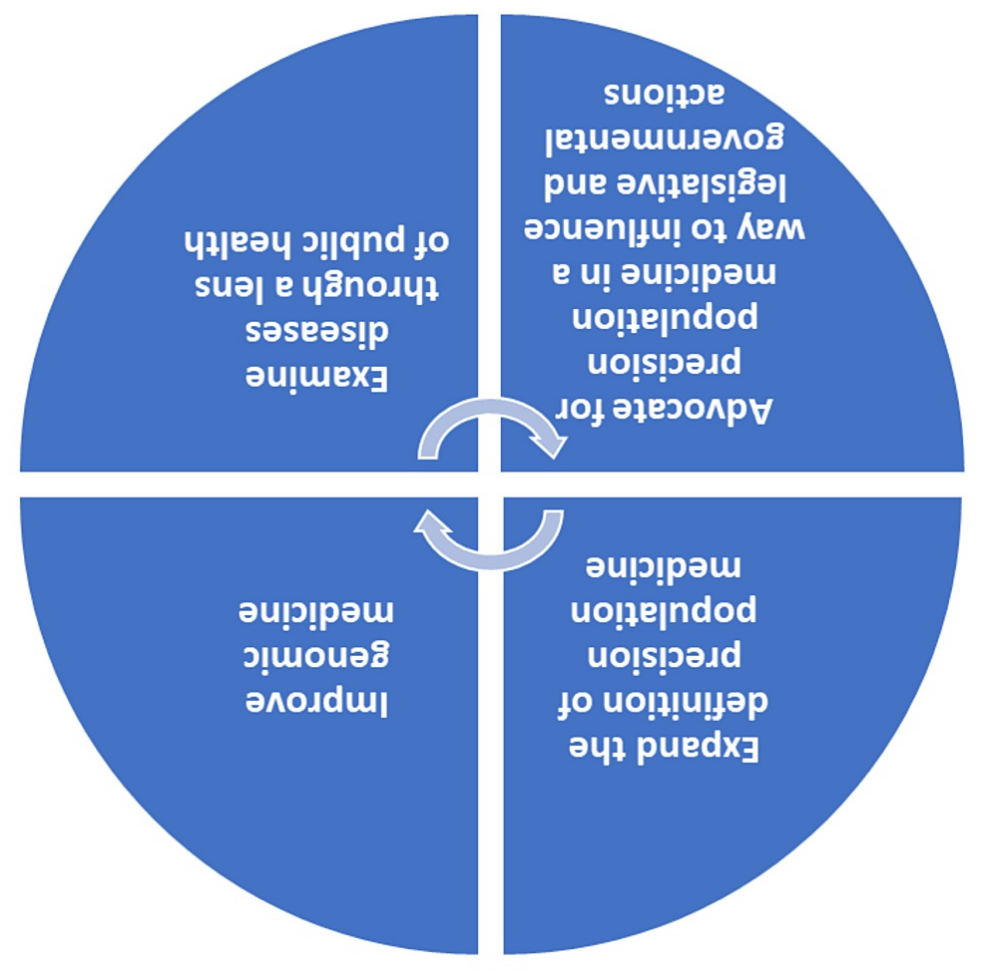
Potential areas for developing precision population medicine. This image is a simplified depiction from the authors of this paper.

Combining GM and PH goals, an expanded definition of PM, or PPM, is significant for breast, cervical, and many more cancers. The applications of liquid biopsies, big data initiatives, MCED, and Galleri can yield substantial improvements in population health, including those in underrepresented populations. Improving equity can be possible, but populations should be analyzed individually. The inception and current developments of PPM motivate to potentially further (a) expand its definition, (b) improve GM, (c) examine diseases with a lens of PH, and (d) advocate for PPM in a way to influence legislative and governmental actions that would help improve the overall health of the populations (Figure 12).

How we intend to prove that “precision population medicine” will help improve health outcomes

Considering population statistics and individual analyses, the new definition of precision medicine will help improve the development of effective treatment plans. When employing some of the previously mentioned tools with the new PM definition, we predict that the desired results will be produced more efficiently when compared to using the previous definition. For example, while liquid biopsies and new-generation sequencing techniques are strategies early in their development, their diagnostic utility and ability to improve patient outcomes serve as the primary motivations to continue their utilization and further investigate their potential. Moreover, our initial priority should aim to encourage population health scientists and policymakers to adopt PPM as a priority (i.e., laws that contrast and highlight the differences in definitions).

Following this, we can consider employing technology developed over these past couple of years to record and analyze our results. For example, devices such as smartwatches and smartphones recognize behavioral patterns, which presents a potentially unique and beneficial opportunity to analyze these data for individuals and populations [25]. Consequently, this robust analysis fosters the necessary credibility of PPM among the academic and healthcare communities while concurrently improving patient outcomes (i.e., patients with cancer are diagnosed sooner). For instance, in the previously mentioned cervical cancer study of African American females and HPV35 associations, the population-level analysis of incidences of HPV-associated cancer aided researchers in distinguishing this population (10%) from the rest of the world (2%) [16]. The collective and concerted efforts of scientists and clinicians to expand and improve the current understanding of PH and PPM, along with the support of policymakers to prioritize and fund the research, are instrumental in utilizing precision medicine to its fullest potential, ultimately focusing on improving patient outcomes and care.

## Conclusions

This paper should serve as a preliminary, broad attempt to elucidate the definition of population precision medicine, describe the shortcomings of the current narrow PM definition, suggest the potential benefits of a more-inclusive definition, and promote the adoption of PPM among lawmakers and the academic community. In addition to the conventional epidemiological and prevention population science principles, the concept of PPM consists of a combination of the following: genomic medicine, big data, EHR, telemedicine, smartphone/personal wearable-based health data collections, and interventions. Future studies plan to investigate PPM further through the specific lens of cancer care for patients (i.e., breast and cervix), legislation, radiomics, and artificial intelligence. This work hopes to provide a review of PM and a call to action to redefine, popularize, and employ PPM with a more significant effort to create a healthier world with more cost-effective, productive, and improved outcome scenarios. Although our work through a series of papers will focus on improving cancer care, similar principles can be applied to non-cancer chronic conditions; however, that is beyond the scope of our current aims.
